# Investigation of MMP-1 genetic polymorphisms and protein expression and their effects on the risk of Kashin-Beck disease in the northwest Chinese Han population

**DOI:** 10.1186/s13018-016-0398-6

**Published:** 2016-05-31

**Authors:** Xiaowei Shi, Aili Lv, Jing Ma, Feng Zhang, Yan Wen, Zengtie Zhang, Xiong Guo

**Affiliations:** Department of Paediatrics, The First Affiliated Hospital of Medical Collage of Xi’an Jiaotong University, Xi’an, Shannxi 710061 People’s Republic of China; School of Public Health, Health Science Center, Key Laboratory of Environment and Gene Related Diseases of Ministry of Education, Key Laboratory of Trace Elements and Endemic Diseases of Ministry of Health, Xi’an Jiaotong University, No. 76 Yanta West Road, Xi’an, 710061 People’s Republic of China; Institute for Endemic Disease Control and Prevention of Qinhai Province, Xining, Qinghai 811602 People’s Republic of China

**Keywords:** Kashin-Beck disease, Matrix metalloproteinase-1, Single nucleotide polymorphisms, Enzyme-linked immunosorbent assay, Immunohistochemistry

## Abstract

**Background:**

The etiology of Kashin-Beck disease (KBD), an endemic osteochondropathy, is largely unknown. Matrix metalloproteinase-1 (MMP-1) plays a central role in the initiation and progression of cartilage destruction; however, no study has reported on the relationship between KBD and MMP-1. This study was to investigate the role of MMP-1 in the pathogenesis and progression of KBD.

**Methods:**

Single nucleotide polymorphism (SNP) genotyping was conducted for 274 KBD cases and 248 healthy controls using the Sequenom MassARRAY system. Additionally, the expression of MMP-1 in the knee articular cartilage of 22 KBD patients and 21 controls was analyzed by immunohistochemistry, and the concentration of MMP-1 in their joint fluid was also measured by enzyme-linked immunosorbent assay (ELISA).

**Results:**

The results showed that two SNPs (rs470221 and rs1144396) had a weak association with increased KBD risk; however, the significance of these results did not survive Bonferroni’s correction. Moreover, the percentages of cells expressing MMP-1 in each layer of cartilage were significantly higher in the KBD group than in the controls (*F* = 11.41–28.31, *P* = 0.002–0.000). The concentration of MMP-1 in KBD joint fluid was significantly higher than that in the controls (*t* = 9.83, *P* < 0.0001).

**Conclusions:**

The increased expression of MMP-1 has a potential effect on the risk of KBD in the northwest Chinese Han population. However, six selected SNPs in the MMP-1 gene might not be useful as significant markers for predicting KBD susceptibility in Chinese Han population. Therefore, future studies in the association of MMP-1 with KBD should focus on other candidate SNPs.

## Background

Kashin-Beck disease (KBD) is a chronic osteochondropathy affecting the bones and joints that is endemic to certain geographical areas. According to the *2010 Health Statistical Year Book of China*, the KBD-affected area in China covers 366 counties of 14 provinces or autonomous regions encompassing more than 690,000 KBD patients and a population of 10.584 million individuals at high risk of developing the disease. A key pathological feature of KBD is chondrocyte necrosis in the deep zone of the growth plate of cartilage and articular cartilage [1, 2]. Clinically, the disease usually presents in childhood, between 5 and 13 years of age, and mainly attacks the growth plate cartilage. KBD presents as dwarfism, very short upper limbs, and deformed and painful joints. The most frequently affected sites are the distal limb joints (e.g., ankles, knees, wrists, and elbows). Following cartilage damage, atrophied muscles occur due to joint pain and limited mobility; these deformed joints make manual farming work more difficult and painful.

The etiology of KBD remains unclear; over the past 150 years, three environmental hypotheses have been proposed: selenium deficiency, mycotoxins from contaminated storage grains, and organic matter (e.g., fulvic acid or FA) in drinking water [3–5]. However, these theories cannot account for the variable differences in the occurrence of KBD among residents exposed to the same environmental risk factors [6]. In our previous study, we found that the incidence of KBD occurred in familial clusters and that 35.10 % of cases could be attributed to the polymorphic variations of KBD [7]. Certain susceptibility genes may affect susceptibility to environmental factors, such as selenium deficiency or other biologic factors [8, 9]. The role of matrix metalloproteinases (MMPs) in the degradative events invoked in the cartilage and bone of arthritic joints has long been appreciated [10, 11]. In a previous association analysis, we indicated that gene markers near locus D11S4094 should be further studied [9]. The gene coding for MMP-1 (11q22-q23) is near D11S4094 (11q23); studies on the MMP family have shown that MMP-1 may play a central role in the initiation and progression of cartilage destruction [12, 13]. However, to the best of our knowledge, no study has reported on the relationship between KBD and MMP-1. In this study, for the first time, we evaluated whether MMP-1 has a potential effect on the risk and progression of KBD.

## Methods

### Study population

In total, 522 unrelated Chinese Han individuals were included in the current study; these individuals were from KBD-endemic areas of the Linyou and Yongshou counties of Shaanxi province, in northwest China. This group consisted of 274 KBD patients and 248 healthy controls. Radiographs of the right hand were taken for both the KBD patients and the healthy controls and read by veteran orthopedists. KBD was diagnosed according to the national diagnostic criteria of China (WS/T 207-2010), which are based on radiographic examination (i.e., an X-ray of the right hand) and clinical diagnosis (from degree I to III). Patients with clinical symptoms or radiographic changes associated with other types of osteochondropathy were excluded. A healthy case was defined as no KBD and no primary or secondary osteoarthritis (OA). The controls were randomly selected and were frequency-matched by age and sex. Fresh blood (5 mL) was collected from the antecubital vein of all 522 subjects while in a fasting state.

Knee articular cartilage and synovial joint fluids were collected from 22 KBD patients and 21 healthy controls. The KBD patients, consisting of ten males and 12 females with an average age of 51.00 ± 8.30 (32–66) years, underwent knee debridement or arthroplasty at a hospital. The healthy control subjects, consisting of 11 males and ten females with an average age of 48.23 ± 7.65 (33–61) years, had no history of osteochondropathy, but they had undergone amputation because of traffic accidents. No significant differences were observed between KBD and control group in age (*t* = 1.13, *P* > 0.05) and sex (*x*^2^ = 0.21, *P* > 0.05). None of the KBD patients or controls was diagnosed with bone or cartilage genetic diseases or rheumatoid arthritis (RA).

The study was performed in accordance with the Declaration of Helsinki and approved by the Human Ethics Committee of Xi’an Jiaotong University, People’s Republic of China. Written informed consent was also obtained from the subjects or their relatives.

### SNPs selection and genotyping

This study evaluated six single nucleotide polymorphisms (SNPs) of MMP-1; we selected SNPs across the gene loci to ensure a high density of markers and to provide an adequate characterization of haplotype diversity. The selected SNPs were required to have an *r*^2^ threshold of 0.8 and minor allele frequency (MAF) ≥5 % in the Chinese Han population [14] (Table 1). Genomic DNA was extracted from the peripheral blood using a blood DNA extraction kit (TIANGEN, Beijing, China). Genotyping was performed using the Sequenom MassARRAY system. Primers were designed using Sequenom SNP Assay Design software version 3.0 for iPLEX reactions. The protocol and reaction conditions were in accordance with the manufacturer [15]. Data management and analysis were conducted by Sequenom Typer 4.0 Software.

**Table 1:** The loci information of the six SNPs in MMP-1

SNPs	Alleles^a^	SNP location	Minor allele frequency (%)			HWE test (*P*)
			KBD	Controls	*P* values	
rs2071231	T/G	Intron	21.5	17.5	0.14	0.99
rs7125062	T/C	Intron	15.1	15.7	0.78	0.97
rs470221	G/A	Intron	33.2	29.8	0.45	0.29
rs470558	G/A	Exon	18.1	16.3	0.47	0.36
rs470206	C/T	5′UTR	21.4	20.4	0.86	0.85
rs1144396	C/A	5′UTR	34.1	31.0	0.37	0.46

*HWE* Hardy-Weinberg equilibrium

^a^Stands for the major/minor alleles

### Immunohistochemistry

The cartilage tissues were immediately fixed in 4 % (*w*/*v*) paraformaldehyde following collection, washed in phosphate-buffered saline (PBS), decalcified, embedded in paraffin, and cut into 5–8-μm-thick slices for immunohistochemistry and hematoxylin and eosin (HE) staining. Immunochemical identification was performed using the streptavidin-peroxidase (SP) method. Briefly, after deparaffinization, endogenous peroxidase was blocked with 3 % H_2_O_2_ for 15 min, after which the slides were washed with PBS. The slides were then predigested using a digestive complex followed by rinsing with PBS. After blocking using 10 % normal goat serum, the sections were incubated with a primary antibody recognizing MMP-1 at 1:100 dilution (polyclonal rabbit anti-MMP-1, Bioss Co, Beijing, China) or with PBS, serving as a negative control, at 4 °C overnight. Next, the sections were incubated with 1:200 biotinylated goat anti-rabbit IgG (ZSGB-Bio Co, Beijing ,China) at 37 °C for 20 min, followed by incubation with horseradish peroxidase-labeled streptavidin solution at 37 °C for 15 min. Color development was continued for 5 min at room temperature using diaminobenzidine followed by rinsing with distilled water. Counterstaining was performed with hematoxylin.

The percentage of positive cells was evaluated by counting within 10 high-magnification power fields (40×) in six consecutive tissue sections. The level of expression was determined using the following semi-quantitative criteria: 0 = negative; 1+ = less than 25 % positive staining; 2+ = 25–50 % positive staining; 3+ = more than 50 % positive staining [1].

### Enzyme-linked immunosorbent assay measurement of MMP-1 in joint fluid

The joint fluid samples were dispensed into 1-ml aliquots that were treated with 50 ug/ml of hyaluronidase for 1 h at room temperature. The joint fluid was then diluted with diluent’s buffer to the appropriate detection range for enzyme-linked immunosorbent assay (ELISA). The MMP-1 level was measured using a commercial ELISA kit (R&D System, Inc 614 McKinley Place NE, Minneapolis, MN 55413, USA) in accordance with the manufacturer’s manual.

### Statistical analyses

The Hardy-Weinberg equilibrium (HWE) of each SNP was tested by the goodness-of-fit *x*^2^ test to compare the expected frequencies of genotypes in controls, SNPs with *P* > 0.05 were considered to be in HWE [16]. Differences in genotypes and allele frequencies between the KBD cases and the controls were determined using CLUMP22 software with 10,000 Monte Carlo simulations. Unconditional logistic regression analysis adjusted for age and gender was used to estimate the strength of the association through calculation of odds ratios (ORs) with their 95 % confidence intervals (95 % CIs) [17]. Dominant and recessive genetic models were also evaluated and expressed as ORs with 95 % CI [18]. To account for multiple testing, Bonferroni’s correction was applied, consisting of dividing the usual significance level of 5 % by the number of tests [17]. Differences in MMP-1 expression in the cartilage between the two groups were examined by analysis of variance (ANOVA) [19]; the independent-sample *t* test was used to compare the concentrations of MMP-1 in joint fluid of the two groups. *P* < 0.05 was considered to indicate statistically significant differences.

## Results

### Baseline characteristics

A total of 274 KBD patients and 248 age and sex matched controls were included in the genetic polymorphism study. No significant differences were observed between KBD and control group in age (53.37 ± 10.79 vs 51.71 ± 17.85, *t* = 1.29, *P* > 0.05) and sex (male/female, 125/149 vs 124/124, *x*^2^ = 1, *P* > 0.05). In addition, the clinical stages of the 274 KBD patients were divided into I, II, and III stages, and the constituent ratio was 57.66 % (158/274), 31.02 % (85/274), and 11.32 % (31/274), respectively.

### Association of polymorphisms of MMP-1 with KBD susceptibility

Table 1 showed the information of the six SNPs, all tested SNPs were in HWE (*x*^2^ = 0.02–2.41, *df* = 2, *P* = 0.29–0.99). When the allele frequencies were compared between the KBD cases and the controls, no significant association was detected in the six SNPs (Table 1). The distribution frequencies of genotypes in rs470221 and rs1144396 were significantly different between the two groups (Table 2). Dominant and recessive models were applied to analyze the association between the polymorphisms and KBD; the ORs and 95 % CIs for KBD, as determined from the unconditional logistic regression models, were specifically used to evaluate the relative risk. The SNPs rs470221 and rs1144396 showed risk association with KBD in the recessive model (Table 2), while the powers were calculated; the results showed that our study had 72.4 and 67.3 % power to detect a nominal significant finding (alpha = 0.05) in the KBD cohort for rs470221 and rs1144396, respectively. However, the significance of these results did not remain after correction for multiple testing (*P* > 0.05/6 = 0.008).

**Table 2:** Analysis of association of the six SNPs gene polymorphism with the risk of KBD

SNPs	Genotype (%)^a^			Recessive model	Dominant model
	KBD (*N* = 274)	Controls (*N* = 248)	*P* values	OR (95 % CI)	OR (95 % CI)
rs2071231	60.6/35.8/3.6	68.1/28.6/3.3	0.19	1.17 (0.43–3.18)	1.40 (0.97–2.02)
rs7125062	73.0/23.7/3.3	71.4/25.8/2.8	0.73	1.36 (0.48–3.87)	0.93 (0.63–1.37)
rs470221	47.8/37.9/14.3	47.6/45.2/7.2	0.02	2.12 (1.18–3.82)	0.99 (0.70–1.40)
rs470558	66.8/30.3/2.9	70.6/26.2/3.2	0.58	0.80 (0.30–2.10)	1.17 (0.87–1.69)
rs470206	60.2/36.5/3.3	63.3/32.7/4.0	0.46	0.65 (0.26–1.65)	1.12 (0.87–1.59)
rs1144396	47.0/37.8/15.2	46.4/45.2/8.4	0.04	1.96 (1.11–3.38)	0.96 (0.68–1.36)

^a^Homozygote of the major allele/heterozygote/homozygote of the minor allele

### Expression of MMP-1 in articular cartilage and levels of MMP-1 in joint fluids of two groups

**Fig. 1:**
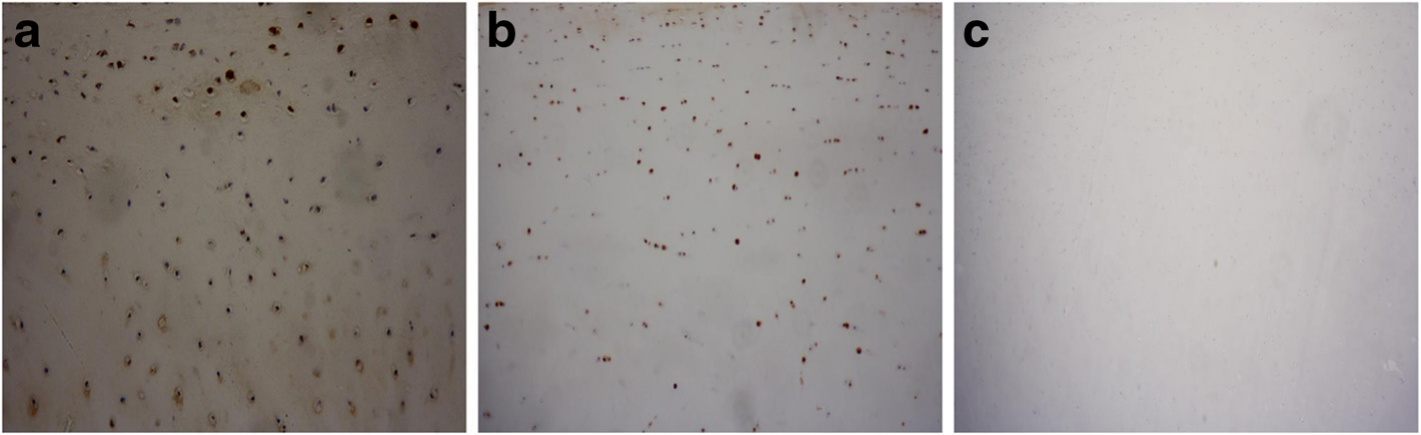
Expression of MMP-1 in different layers of cartilage (immunohistochemistry using the SP method; original magnification ×100). **a** The cartilage of a KBD. **b** The cartilage of a healthy control. **c** A negative control with PBS was used instead of the anti-MMP-1 primary antibody

Microscope observation revealed that MMP-1 staining was distributed throughout all zones in the articular cartilages of KBD patients and healthy controls. Starting from the uppermost layer, the percentage of chondrocytes showing MMP-1 staining decreased in each successive layer of cells. In contrast, in the negative-control articular cartilage, MMP-1 was not visualized (Fig. 1). A summary of the levels of MMP-1 expression in cartilage from KBD patients and controls is presented in Table 3. In each layer of the articular cartilage, the KBD cases showed a higher level of MMP-1 positive staining (Fig. 1, Table 3). A comparison of the positive rate of MMP-1 stained cells in different layers between the two groups showed that the percentages of stained cells in the KBD group were significantly higher than those in the controls in the upper, middle, and deeper layers (*F* = 14.98, 11.41, 28.31, respectively; all *P* < 0.05).

**Table 3:** Immunohistochemical expression of MMP-1 in different layers of the articular cartilage in the two groups

Articular cartilage	Groups	Level of expression				Positive rate (%)
		Negative	+	++	+++	Mean ± SD
Upper layer	Control (*n* = 21)	0	0	13	8	45.27 ± 20.48
	KBD (*n* = 22)	0	0	3	19	67.02 ± 16.19*
Middle layer	Control (*n* = 21)	0	9	7	5	35.71 ± 24.80
	KBD (*n* = 22)	0	1	6	15	58.19 ± 18.01*
Deeper layer	Control (*n* = 21)	6	15	0	0	3.00 ± 32.44
	KBD (*n* = 22)	0	9	8	5	34.87 ± 27.23*
Total layers	Control (*n* = 21)	0	12	7	2	27.92 ± 13.59
	KBD (*n* = 22)	0	0	11	11	53.32 ± 14.58*

*P < 0.05 compared with the control group

**Fig. 2:**
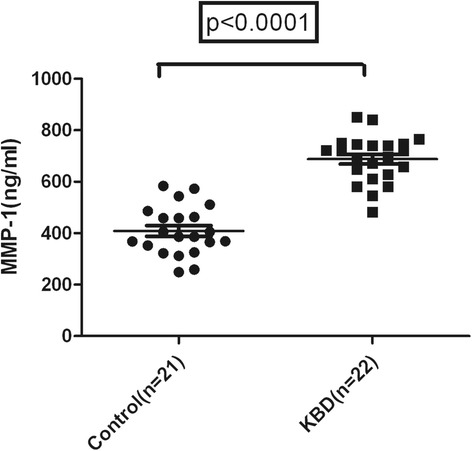
MMP-1 concentrations in synovial fluids of knees from KBD and controls. Levels of MMP-1 in KBD samples were significantly higher than those in controls. Statistical significance was determined using *t* test. Result was considered to be significant at *P* < 0.05

The joint fluid was measured by ELISA; MMP-1 levels in KBD joint fluid were significantly higher than those in the controls (Fig. 2). The mean ± SEM levels of MMP-1 in the joint fluids of KBD and controls were 687.95 ± 90.92 ng/ml (482–841 ng/ml) and 408.67 ± 95.31 ng/ml (249–573 ng/ml), respectively (*t* = 9.83, *P* < 0.0001).

## Discussion

KBD is an endemic OA with a specific geographical distribution. However, not all families or individuals in endemic areas suffer from KBD. Additionally, recent studies reported that susceptibility genes might contribute to the pathogenesis of KBD and account for an individual’s susceptibility to KBD [20]. MMP-1 is the most highly expressed interstitial collagenase that degrades fibrillar collagens, which are major constituents of the extracellular matrix (ECM) [21]. Many studies have provided evidence for an association between the MMP-1 gene and arthritis [22, 23]. KBD has been found to have overlapping phenotypes and pathologic changes similar to those of OA and RA [24]. The polymorphism study of KBD is very limited; a similar study showed a significant association between rs6910140 of COL9A1 and KBD [15]. Used genome-wide association study (GWAS), ITPR2, was identified as a susceptibility gene for KBD [25]. However, none of the six selected SNPs in the MMP-1 gene showed an association with KBD in our study; the finding of this study was not consistent with results of previous studies, and we think some limitations should be considered in relation to present results: First, it is common for a genetic variant to be associated with certain populations but not with others [26], and we failed to investigate the role of MMP-1 in the pathogenesis and progression of KBD in different populations. Second, the number of SNPs was very limited in this study; the susceptibility of KBD may be associated with other SNPs in the MMP-1 gene. So we think it should not dismiss a possible association between MMP-1 gene polymorphism and KBD; the gene’s roles in determining the expression of MMP-1 should be further studied, and an approach examining the common genetic variation of the gene should be used.

MMPs are involved in joint destruction in arthritis and are strongly associated with the levels of inflammation. MMP-1 is more abundant and also degrades collagens effectively. In the present study, the expression of MMP-1 in articular cartilage of knee was analyzed by immunohistochemistry; the negative control showed negative staining, which indicated that the test was not confounded by other factors and was reasonable. Our results showed increased expression of MMP-1 should play a role in the pathogenesis of KBD. These results are similar to those found in other degenerative joint diseases, such as OA and RA. Regarding KBD, a similar study has been reported, in which human chondrocytes were isolated and cultured on bone matrix gelatin to form an artificial cartilage model in vitro with or without T-2 toxin and selenium. With exposure to the KBD risk factors (with T-2 toxin and without selenium), Western blotting and RT-PCR analyses showed level of MMP-1 was increased significantly after exposure of T-2 toxin; increasing doses of T-2 toxin resulted in an increased expression of MMP-1; selenium increased TIMP-1 expression and decreased MMP-1 expression and partly blocked the effects of T-2 toxin on the balance between TIMP-1 and MMPs [27]. More specifically, studies on the expression of MMP-1 in patients with RA or OA found that the circulating levels of MMP-1 were elevated. In our study, the levels of MMP-1 in joint fluid of knee were also detected and the data showed higher level of MMP-1 in patients with KBD than in controls. A previous study in the cases of the same KBD area showed that the serum MMP-1 levels in KBD cases were higher than those of controls, even though no significant difference was detected [1]. All these studies showed that elevated levels of MMP-1 in cartilage and synovium may be a cause of degrading cartilage in the pathogenesis of KBD in Chinese Han population.

Tight regulation of MMP activity is critical for maintaining a fine balance between aggrecan and collagen anabolism and catabolism. The MMP-1 enzyme is known as collagenase or collagenase-1, and it degrades a broad range of substrates (e.g., collagens I, II, III, VII, VIII, and X; gelatin; aggrecan; versican; and proteoglycan link protein), leading to collagen network edema that can be inhibited by TIMP-1. Articular cartilage contains a large number of matrix macromolecules, such as proteoglycan, aggrecan, and type II collagen [28, 29], which provide elastic and strong tensile properties that resist mechanical loading. Under normal conditions, there is a balance between catabolism and anabolism in the ECM in articular cartilage [30, 31]. However, certain conditions, such as inflamed synovium and pannus tissue, may disturb this balance and accelerate catabolism of the ECM, leading to loss of ECM components from the cartilage, thereby severely impairing ECM functionality and causing pain and disability [32]. Studies have reported that IL-1β and INF-α expression in the synovium, and their levels in synovial fluid and serum are significantly higher in patients with KBD than in controls [1, 33]. We think that MMP-1 overexpression induced by IL-1β, INF-α, and other inflammation factors may play an important role in destroying the articular cartilage of KBD patients.

However, there are also some limitations to this study: the sample populations were a little small and only MMP-1 was included in this study. And it has been reported that selenium has a protective role while T-2 toxin induces cartilage matrix degradation by regulation of MMP-1 /TIMP-1 expression; however, environmental factors such as low Se and T-2 toxin were not analyzed in this study. Therefore, the effect of gene-environment interactions needs further investigation in future well-designed high-quality studies among well-subgrouped populations.

## Conclusions

In conclusion, up-regulation of MMP-1 in the ECM accelerates ECM catabolism, leading to matrix degradation, cartilage degeneration, and subsequent pathogenesis and progression of KBD. However, the six selected SNPs in the MMP-1 gene might not be useful as significant markers for predicting KBD susceptibility in the northwest Chinese Han population. Therefore, analysis of the genetic component of KBD needs further investigation.
